# Antioxidant Effect of Melatonin on the Functional Activity of Colostral Phagocytes in Diabetic Women

**DOI:** 10.1371/journal.pone.0056915

**Published:** 2013-02-20

**Authors:** Gliciane Morceli, Adenilda C. Honorio-França, Danny L. G. Fagundes, Iracema M. P. Calderon, Eduardo L. França

**Affiliations:** 1 Post Graduate Program in Gynecology, Obstetrics and Mastology of Botucatu Medical School, São Paulo State University/Unesp, Botucatu, São Paulo, Brazil; 2 Institute of Biological and Health Science, Federal University of Mato Grosso, Barra do Garças, Mato Grosso, Brazil; Children's Hospital Boston, United States of America

## Abstract

Melatonin is involved in a number of physiological and oxidative processes, including functional regulation in human milk. The present study investigated the mechanisms of action of melatonin and its effects on the functional activity of colostral phagocytes in diabetic women. Colostrum samples were collected from normoglycemic (N = 38) and diabetic (N = 38) women. We determined melatonin concentration, superoxide release, bactericidal activity and intracellular Ca^2+^ release by colostral phagocytes treated or not with 8-(Diethylamino) octyl-3,4,5-trimethoxybenzoate hydrochloride (TMB-8) and incubated with melatonin and its precursor (N-acetyl-serotonin-NAS), antagonist (luzindole) and agonist (chloromelatonin-CMLT). Melatonin concentration was higher in colostrum samples from hyperglycemic than normoglycemic mothers. Melatonin stimulated superoxide release by colostral phagocytes from normoglycemic but not hyperglycemic women. NAS increased superoxide, irrespective of glycemic status, whereas CMTL increased superoxide only in cells from the normoglycemic group. Phagocytic activity in colostrum increased significantly in the presence of melatonin, NAS and CMLT, irrespective of glycemic status. The bactericidal activity of colostral phagocytes against enterophatogenic *Escherichia coli* (EPEC) increased in the presence of melatonin or NAS in the normoglycemic group, but not in the hyperglycemic group. Luzindole blocked melatonin action on colostrum phagocytes. Phagocytes from the normoglycemic group treated with melatonin exhibited an increase in intracellular Ca^2+^ release. Phagocytes treated with TMB-8 (intracellular Ca^2+^ inhibitor) decreased superoxide, bactericidal activity and intracellular Ca^2+^ release in both groups. The results obtained suggest an interactive effect of glucose metabolism and melatonin on colostral phagocytes. In colostral phagocytes from normoglycemic mothers, melatonin likely increases the ability of colostrum to protect against EPEC and other infections. In diabetic mothers, because maternal hyperglycemia modifies the functional activity of colostrum phagocytes, melatonin effects are likely limited to anti-inflammatory processes, with low superoxide release and bactericidal activity.

## Introduction

Diabetes is prevalent in young women and is increasingly related to maternal and child health issues, such as breastfeeding. Several studies have investigated the association of breastfeeding with a variety of chronic diseases, including obesity and diabetes [1], [2]. Infants born to diabetic women are at higher risk of hypoglycemia because maternal hyperglycemia causes fetal hyperinsulinism [3]. However, the impact of breastfeeding on glucose metabolism is only partially understood.

Breastfeeding decreases the risk of diabetes development [4], infant morbidity and mortality and prevents gastrointestinal and respiratory infections [5], [6], [7], [8], [9]. Human milk contains soluble and cellular components such as lipids, carbohydrates, proteins, cells and hormones, which are important for the nutrition and immunological defense of infants [10]. Melatonin (MLT), one of the hormones contained in milk, is produced by the pineal gland and plays an important for infants [11].

The benefits of MLT and its metabolites are related to their antioxidant and anti-inflammatory properties [12], [13] and prooxidant effects [14]. MLT also affects glucose regulation in humans [15], [16]. Diabetic patients have lower diurnal serum MLT levels and more pancreatic melatonin-receptors [17], [18]. The role of MLT in preventing or delaying diabetes onset, however, is not well established, because studies showing the beneficial effects of MLT have been conducted after the development of the clinical manifestation of diabetes [19], [20]. In addition, the actions of MLT on endocrine pancreas physiology, including the probable reduction in diabetes incidence, have not been well described [18].

MLT is not a conventional hormone because it displays both receptor-mediated and receptor-independent actions. Therefore, regardless if they possess indolamine receptors, all cells in the body are a target for melatonin. MLT interacts with membrane and nuclear receptors. Membrane receptors mediate functions such as seasonal reproduction, sleep and bone growth [16]. Using receptor-mediated and receptor independent mechanisms, melatonin seems to be involved in a number of physiological and metabolic processes. MLT can affect the levels of 3′-5′-cyclic adenosine monophosphate (cAMP) and intracellular Ca^2+^, and its action seems to be transmitted through the Gq-coupled membrane receptor action on phospholipase C (PLC) and inositol triphosphate (IP3) [21].

Human colostrum also contains large amounts of viable leukocytes, most of them macrophages. Both circulating and colostral leukocytes have phagocytic and bactericidal activity and produce free radicals [5], [8], [22], [23]. Macrophages in diabetic patients were shown to have lower phagocytic and microbicidal activity and lower production of reactive oxygen species [8], [24], [25]. This suggests that the antioxidant systems of diabetic individuals are likely compromised by high glucose levels [25]. Despite this evidence, studies on the functional activity of phagocytes in the colostrum of diabetic mothers, as well as the action of these defense cells on the gut of newborns and their interactions with melatonin are scarce. It is known that colostrum components possibly play an important protective role against pathogens in newborn guts, and colostral melatonin [11] may directly affect many gastrointestinal tissues. In addition to stimulating the immune system, melatonin prevents ulcerations of the gastrointestinal mucosa by antioxidant action [26].

The biological activity of colostral MLT and its interactions with milk components is important because colostrum consists of a complete micro-environment, where the combined action of soluble and cellular components possibly plays a significant protective role against pathogens in newborn guts, especially considering infants of diabetic mothers who are highly susceptible to infections. Given the lack of information on this issue, the present study investigated the mechanisms of action of MLT and its effects on the functional activity of phagocytes in the colostrum of diabetic women.

## Materials and Methods

The effect of melatonin on the functional activity of colostrum phagocytes in hyperglycemic women was evaluated in a cross-sectional study. The subjects attended the Diabetes and Pregnancy Facility, School of Medicine Obstetrics Course, UNESP, Botucatu, SP, Brazil.

### Ethics Statement

This study was approved by the institutional Research Ethics Committee of Botucatu Medical School, and all the subjects gave informed written consent before entering the experimental protocol.

### Subjects

We evaluated 76 women (18–45 years old) tested for hyperglycemia between the 24^th^ and 28^th^ weeks of pregnancy. Hyperglycemia was diagnosed by the 100 g – oral glycemia tolerance test (OGTT test), according to American Dietetic Association criteria [27], and the glucose profile (GP) test, according to Gillmer's threshold values [28]. After delivery, colostrum samples from these subjects were analyzed according to maternal glycemic status: normoglycemic group (normal 100 g-OGTT and normal GP; n = 38) and diabetes group (abnormal pre-pregnancy 100 g-OGTT and insulin-dependent; n = 38). The mean and standard deviation for gestacional age were 38.9±1.1 weeks in normoglycemic and the 37.3±0.9 weeks in diabetes groups and newborns' birth weight (g) were 3127.3±339.4 in normoglycemic and the 3266.3±469.8 in diabetes groups. The subjects continued attending the facility, irrespective of diagnosis, and the hyperglycemic patients followed a specific treatment for glycemic control [28].

The variables controlled were smoking status (yes/no), arterial hypertension (yes/no) and glycemic index (GI), whose mean plasma glucose level was measured in the glycemic profiles taken during the gestation. GI was classified as adequate (GI<120 mg/dL) or inadequate (GI≥120 mg/dL) [28].

### Colostrum sampling and separation of colostral cells

About 8 mL of colostrum from each woman was collected in sterile plastic tubes between 48 and 72 hours postpartum. The samples were centrifuged (160× g, 4°C) for 10 min, which separated colostrum into three different phases: cell pellet, an intermediate aqueous phase, and a lipid-containing supernatant. The upper fat layer was discarded and the aqueous supernatant stored at −80°C for later analyses. Cells were separated by a Ficoll-Paque gradient (Pharmacia, Upsala, Sweden), producing preparations with 98% of pure mononuclear cells, analyzed by light microscopy. Purified macrophages were resuspended independently in serum-free medium 199 at a final concentration of 2× cells/mL.

### Melatonin hormone dosage by the immunoenzymatic method

MLT was extracted by affinity chromatography, concentrated by speed-vacuum and determined using the ELISA kit (Immune-Biological Laboratories, Hamburg, Germany) [29]. Reaction rates were measured by absorbance plate-reading spectrophotometer with a 405 nm filter. The results were calculated according to the standard curve and shown in pg/mL.

### Treatment of colostral mononuclear phagocytes with melatonin and its precursor, antagonist and agonist

To assess the effects of melatonin on superoxide anion release, as well as on phagocytic and microbicidal activity, mononuclear phagocytes (2×10^6^cells/mL) were incubated with 50 µL MLT (Sigma, ST Loius, USA; at a final concentration of 10^−7^ M [30], 50 µL N-acetyl-serotinin (NAS - Sigma, ST Loius; USA at a final concentration of 10^−7^ M), 50 µL luzindole (Sigma, ST Loius, USA; at a final concentration of 10^−7^ M and 50 µL chloromelatonin (CMLT – Sigma, ST Loius, USA; at a final concentration of 10^−7^ M) for 1 h at 37°C.

To investigate the effects of intracellular Ca^2+^ on MLT action, phagocytes (2×10^6^ cells/mL) were incubated with 10 µL of 8-(Diethylamino)octyl-3,4,5-trimethoxybenzoate hydrochloride (TMB-8 intracellular calcium inhibitor - at a final concentration of 0.1 mM, Sigma, ST Loius, USA) for 1 h at 37°C. The phagocytes were then washed once with medium 199 at 4°C and immediately used in the assays developed to measure superoxide release, phagocytosis and bactericidal activity.

### Escherichia coli strain

The enterophatogenic *Escherichia coli* (EPEC) used was isolated from stools of an infant with acute diarrhea (serotype 0111:H2, LA1, eae1, EAF1, bfp1). This material was prepared and adjusted to 10^7^ bacteria/ml, as previously described [22].

### Release of superoxide anion

Superoxide release was determined by cytochrome C (Sigma, ST Loius, USA) reduction [22], [31]. Briefly, mononuclear phagocytes and bacteria, opsonized or not with the aforementioned opsonins, were mixed and incubated for 30 min for phagocytosis. Cells were then resuspended in Phosphate Buffer Solution (PBS) containing 2.6 mM CaCl_2_, 2 mM MgCl_2_, and cytochrome C (Sigma, ST Loius, USA; 2 mg/mL). The suspensions (100 µL) were incubated for 60 min at 37°C on culture plates. The reaction rates were measured by absorbance at 550 nm and the results were expressed as nmol/O_2_^−^. All the experiments were performed in duplicate or triplicate.

### Bactericidal assay

Phagocytosis and Microbicidal activity were evaluated by the acridine orange method described by Bellinati-Pires et al. [32]. Equal volumes of bacteria and cell suspensions were mixed and incubated at 37°C for 30 min under continuous shaking. Phagocytosis was stopped by incubation in ice. To eliminate extracellular bacteria, the suspensions were centrifuged twice (160× *g*, 10 min, 4°C). Cells were resuspended in serum-free medium 199 and centrifuged. The supernatant was discarded and the sediment dyed with 200 µL of acridine orange (Sigma, ST Loius, USA; 14.4 g/L) for 1 min. The sediment was resuspended in cold culture 199, washed twice and observed under immunofluorescence microscope at 400× and 1000× magnification. The phagocytosis index was calculated by counting the number of cells ingesting at least 3 bacteria in a pool of 100 cells. To determine the bactericidal index, we stained the slides with acridine orange and counted 100 cells with phagocytized bacteria. The bactericidal index is calculated as the ratio between orange-stained (dead) and green-stained (alive) bacteria ×100 [8]. All the experiments were performed in duplicate.

### Intracellular Ca^2+^ release determined by fluorescence and flow cytometry

We performed fluorescence staining at the FACS Calibur (BD San Jose USA) to assess intracellular Ca^2+^ release in colostrum phagocytes [33]. Cells were loaded with the fluorescent radiometric calcium indicator Fluo3-Acetoxymethyl (Fluo3-AM– Sigma ST Louis, USA). Cell suspensions, pre-treated or not with melatonin and TMB-8, mixed and incubated at 37°C for 30 min under continuous stirring. Suspensions were centrifuged twice (160× g, 10 min, 4°C) and resuspended in PBS containing BSA (5 mg/mL). This suspension was incubated with 5 µL of Fluo-3 (1 µg/mL) for 30 min at 37°C. After incubation, cells were washed twice in PBS containing BSA (5 mg/mL; 160× g, 10 min, 4°C) and then analyzed by flow cytometry. Calibration and sensitivity were routinely checked using CaliBRITE 3 Beads (BD Cat. No 340486 USA). Fluo-3 was detected at 530/30 nm filter for intracellular Ca^2+^. The rate of intracellular Ca^2+^ release was expressed in geometric mean fluorescence intensity of Fluo-3. Data shown in the figures correspond to one of several trials performed.

### Statistical analysis

Analysis of variance (ANOVA) was used to evaluate superoxide anion release, phagocytosis, bactericidal index and calcium release. Statistical significance was considered for a p-value of less than 0.05.

## Results

### MLT concentration in colostrum

**Figure 1:**
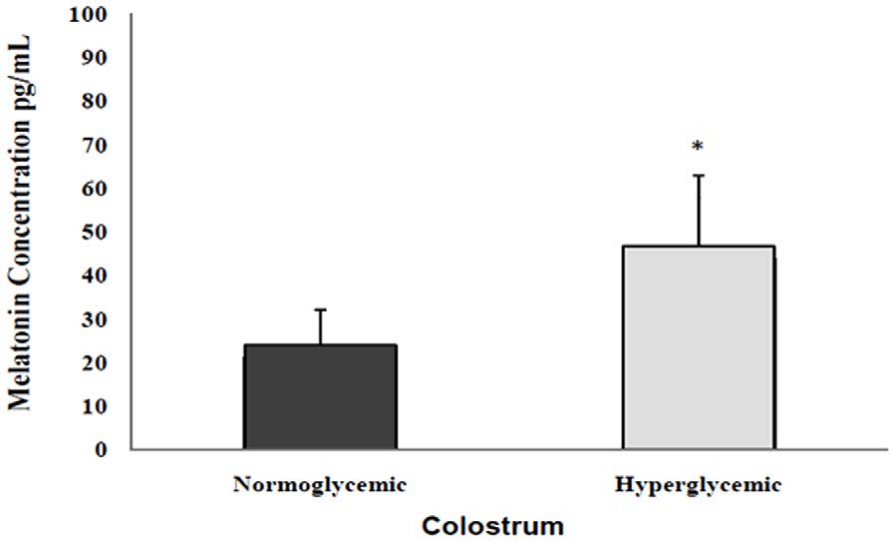
Mean (±SD; N = 10) melatonin levels (pg/mL) in the supernatant of colostrum from diabetic mothers *indicates difference between normoglycemic and hyperglycemic groups.

MLT concentration was higher in colostrum samples from hyperglycemic than normoglycemic mothers (P<0.05; Figure 1).

### Effects of MLT on superoxide release by colostrum phagocytes

Hyperglycemic and normoglycemic groups exhibited similar spontaneous superoxide release by colostral mononuclear phagocytes, which did not increase with phagocyte exposure to EPEC. In the normoglycemic group, phagocytes stimulated with MLT and incubated with EPEC had higher superoxide release than those exposed to the bacteria alone (p<0.05), but MLT stimulation was not observed in the hyperglycemic group. Superoxide release increased when colostral phagocytes were incubated with NAS, irrespective of the group's glycemic status, whereas when incubated with CMTL this anion increased only in the normoglycemic group. Luzidole decreased superoxide release in both groups (Table 1). Phagocytes treated with TMB-8 (inhibitor of intracellular Ca^2+^) displayed a reduction in superoxide release in all the groups studied (Table 1).

**Table 1:** Superoxide release by colostrum phagocytes (mean ± SD, N = 8 per treatment).

Phagocytes incubated with:	TMB-8	Superoxide release (nmol)	
		Normoglycemic	Diabetes
Control (without EPEC)	No	1.2±0.4	1.2±0.1
	Yes	0.5±0.2#	0.4±0.2#
EPEC+PBS	No	1.7±0.6	1.4±0.1
	Yes	0.6±0.3#	0.3±0.1#
EPEC+MLT	No	2.8±0.6*	1.6±0.5+
	Yes	0.2±0.1#	0.2±0.1#
EPEC+NAS	No	2.9±0.4*	2.3±0.4*
	Yes	0.15±0.1#	0.3±0.12#
EPEC+Luzindole+MLT	No	0.5±0.13*	0.40±0.05*
	Yes	0.1±0.02#	0.45±0.03
EPEC+CMLT	No	1,95±0.4*	1.43±0.5
	Yes	0.68±0.2#	1.0±0.3

Phagocytes were incubated with enterophatogenic *Escherichia coli* (EPEC) in the presence of melatonin (MLT), N-acetyl-serotonin (NAS), luzindole or chloromelatonin (CMLT) and pre-treated or not with 8-(Diethylamino) octyl-3,4,5-trimethoxybenzoate hydrochloride (TMB-8). In controls assays, phagocytes were pre-incubated with phosphate buffer solution (PBS).

* indicates difference from the control treatment (ANOVA, P<0.05);

+ indicates intergroup differences within each treatment (ANOVA, P<0.05);

# indicates differences between TMB-8 use within each treatment and group.

### Effects of melatonin on the phagocytic activity of colostral mononuclear cells

**Figure 2:**
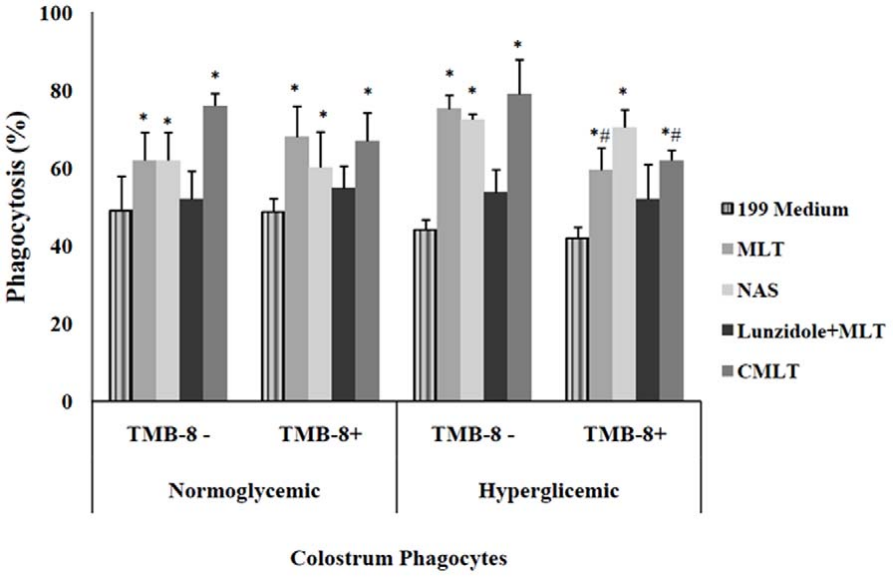
Bacterial phagocytosis by colostral phagocytes (mean ± SD, N = 7 in each treatment), determined by the acridine orange method. Phagocytes were pre-treated or not with 8-(Diethylamino) octyl-3,4,5-trimethoxybenzoate hydrochloride (TMB-8) and incubated with enterophatogenic *Escherichia coli* (EPEC) in the presence of melatonin (MLT), N-acetyl-serotonin (NAS), luzindole or chloromelatonin (CMLT). * indicates differences from the 199 medium (ANOVA, P<0.05); ^#^ indicates differences between TMB-8 use within each treatment and group.

Colostral mononuclear phagocytes from the hyperglycemic groups had some phagocytic activity in response to EPEC. Phagocytosis increased significantly in the presence of MLT. The highest phagocytosis rates for EPEC were also exhibited by colostral phagocytes incubated with NAS and CMLT, independently of glycemic status (Figure 2). Luzindole blocked MLT action on colostral phagocytes. The phagocytosis indexes of cells incubated with luzindole plus MLT were similar to those exhibited by phagocytes incubated with bacteria alone (Figure 2). Pre-treatment of mononuclear phagocytes with TMB-8 decrease the phagocytic activity for EPEC in the diabetic groups treated with MLT and CMLT (Figure 2).

### Effect of melatonin on the bactericidal activity of mononuclear phagocytes

**Figure 3:**
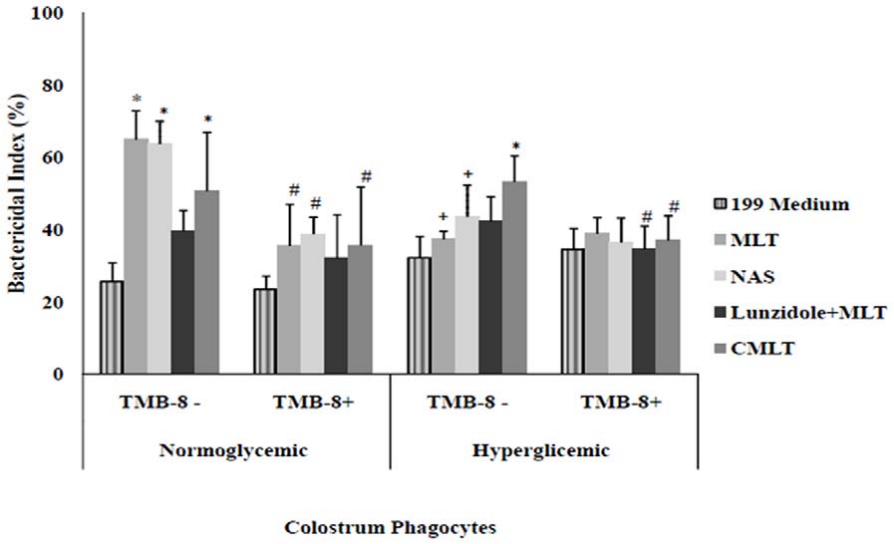
Bactericidal index (mean ± SD, N = 7 in each treatment). Bactericidal activity by colostral phagocytes was determined with the acridine orange method. Phagocytes were pre-treated or not with 8-(Diethylamino) octyl-3,4,5-trimethoxybenzoate hydrochloride (TMB-8) and incubated with enterophatogenic *Escherichia coli* (EPEC) in the presence of melatonin (MLT), N-acetyl-serotonin (NAS), luzindole or chloromelatonin (CMLT). * indicates difference from the 199 medium (ANOVA, P<0.05); + indicates intergroup differences within each treatment (ANOVA, P<0.05); ^#^ indicates differences between TMB-8 use within each treatment and group.

In general, colostral mononuclear phagocytes not treated with MLT had low bactericidal activity against EPEC. In the normoglycemic group, the bactericidal activity was higher when the phagocytes were incubated with MLT or NAS, but this effect was not observed in the hyperglycemic group. Incubation with luzindole decreased the effects of MLT on the bactericidal activity of colostral phagocytes. The bactericidal index of phagocytes incubated with luzindole plus MLT was similar to that of phagocytes incubated with bacteria alone (Figure 3).

After incubation with MLT, NAS and CMLT, mononuclear phagocytes pretreated with TMB-8 had lower bactericidal activity in the normoglycemic groups (Figure 3).

### Intracellular Ca^2+^ release by colostral phagocytes in the presence of melatonin

**Figure 4:**
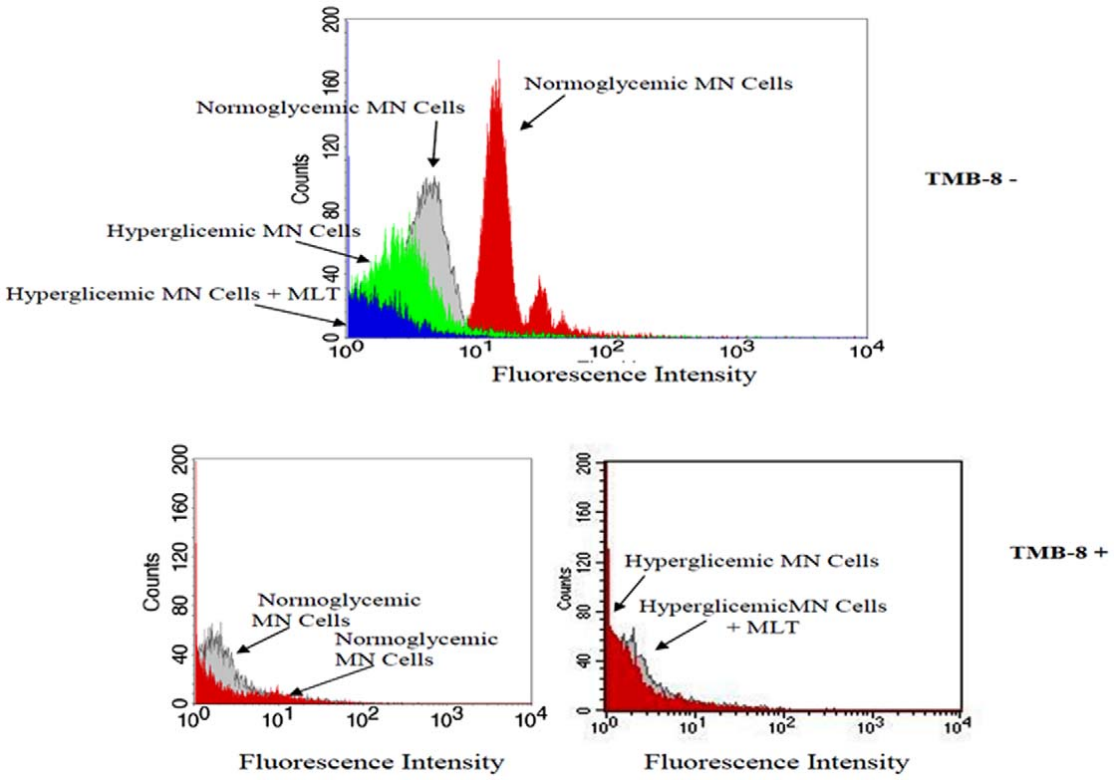
Intracellular Ca^2+^ release by colostral phagocytes from diabetic mothers pre-treated or not with 8-(Diethylamino) octyl-3,4,5-trimethoxybenzoate hydrochloride (TMB-8) and stimulated with melatonin (MLT). Cells were stained with Fluo-3, and immunofluorescence analyses carried out by flow cytometry (FACScalibur, Becton Dickinson, USA).

Mononuclear phagocytes had low spontaneous intracellular Ca^2+^ release. In response to MLT, NAS and CMLT, colostral phagocytes displayed increased intracellular Ca^2+^ release in the normoglycemic groups (Table 2). Colostral from diabetes mothers decrease intracellular Ca^2+^ release in the presence of MLT, NAS and CMLT when compared with the normoglycemic mothers (Figure 2). Incubation with luzindole decreased the effects of MLT on the intracellular Ca^2+^ release (Table 2). Pretreatment of mononuclear phagocytes with TMB-8 decreased intracellular Ca^2+^ release in both groups (Table 2 – Figure 4).

**Table 2:** Intracellular Ca^2+^ release by mononuclear (MN) colostrum phagocytes from diabetic mothers indicated by fluorescence intensity.

Phagocytes	TMB-8	Intensity (%)	
		Normoglycemic (N = 5)	Diabetes (N = 5)
FLUO3-AM	No	35.7±10.2	29.4±8.0
	Yes	4.0±0.6#	5.1±0.8#
MLT+FLUO3-AM	No	64.9±8.0	30.2±5.0+
	Yes	18.4±6.1#	13.0±6.0#
NAS+FLUO3-AM	No	61.4±9.3	38.5±9.7+
	Yes	14,5±7.9#	9.6±3.9#
Luzindole+MLT+FLUO3-AM	No	13.5±2.9*	4.5±1.3* +
	Yes	10.2±5.2	3.9±1.1+
CMLT+FLUO3-AM	No	58.9±12.5	12.3±2.5* +
	Yes	7.5±4.7#	7.7±3.2#

Phagocytes were pre-treated or not with 8-(Diethylamino) octyl-3,4,5-trimethoxybenzoate hydrochloride (TMB-8) and incubated with melatonin (MLT), N-acetyl-serotonin (NAS), luzindole or chloromelatonin (CMLT) and loaded with the fluorescent radiometric calcium indicator FLUO3-AM (Fluo3-Acetoxymethyl) as described in Materials and Methods. Results are expressed as mean and SD (N = 5 per treatment).

* indicates difference from the FLUO3-AM (ANOVA, P<0.05);

+ indicates intergroup differences within each treatment (ANOVA, P<0.05);

# indicates differences between TMB-8 use within each treatment and group.

## Discussion

The major findings of the present study are that MLT was shown to increase superoxide production and bactericidal activity of colostral phagocytes from normoglycemic women, but not colostral phagocytes from hyperglycemic women. The bactericidal activity of colostral cells incubated with NAS, a melatonin precursor, was potentiated in normoglycemic group. Similar results were obtained when phagocytes were exposed to melatonin agonists, suggesting that MLT and its agonist have the same mechanism of action. Finally, the mechanism of action of MLT was shown to be dependent on intracellular Ca^2+^ release.

The beneficial actions of MLT are associated to its ability to scavenge free radicals and increase antioxidant enzyme activity [34], [35], [36]. On the other hand, immune cells produce a high amount of superoxide radical anion during oxidative stress [37], [38], [39], an important protective mechanism during infectious processes, particularly intestinal infections [5], [6], [7], [8], [9], [14], [22], [40], [41]. In the present study, melatonin potentiated the production of these free radicals by phagocytes from normoglycemic mothers. Therefore, colostral MLT probably enhances the protective role of colostrum against EPEC and other infections.

MLT also exhibits immunomodulatotory effects [42] and stimulates immune cells [43], [44]. In this study MLT stimulated the functional activity of phagocytes in the normoglycemic group, increasing EPEC killing capacity. However, an important finding in the present study was that colostral phagocytes from diabetic women were not stimulated by MLT, showing low superoxide release and bacteria killing capacity. The insufficient stimulation of the functional activity of colostral macrophages, e.g., a failure of the prooxidant effect, indicates a diabetes-related antioxidant effect [13]. Similar results were observed in alloxan-induced diabetes models [30], [45], [46], [47], [48].

NAS effects on superoxide release, phagocytic activity and the bactericidal capacity of colostral phagocytes was also evaluated in order to investigate the mechanisms of melatonin action. Interestingly, that NAS also was able to potentiate the bactericidal activity of colostral phagocytes from normoglycemic mothers, but not those from hyperglycemic mothers. On the other hand, the agonist CMLT was able to stimulate the functional activity of colostrum phagocytes, irrespective of glycemic status.

MLT plays a crucial role in a number of metabolic functions such as antioxidant and anti-inflammatory responses [15]. As a multitasking indolamine, MLT seems to be involved in several physiological and metabolic processes via receptor-mediated and receptor-independent mechanisms [49].

To investigate whether MLT exerted its effect upon colostral phagocytes activity through membrane-bound receptors, we used the non-specific MLT receptor antagonist – luzindole that inhibited the effects of hormone. MLT-binding sites have been identified in several human immune cells such as T lymphocytes [50], bone marrow cells and Th2 lymphocytes [51], platelets [52], neutrophils, granulocytes [53] and monocytes [54]. In mammals, MLT activates at least three distinct high-affinity receptors (MT). All three MLT receptors are blocked by luzindole [55].This inhibitory effect was observed in the present study, where the luzindole concentration used blocked MT receptors, thereby decreasing superoxide release and EPEC killing by colostrum phagocytes.

When MLT interacts with its receptors, the latter signal to second messenger 3′-5′-cyclic adenosine monophosphate (cAMP) or inositol triphosphate (IP3)/calcium (Ca2þ), changing intracellular concentrations of either cAMP or calcium. In this study, pretreatment of colostral phagocytes with TMB-8 (intracellular calcium inhibitor) showed that the mediation of phagocytic activity by MLT is calcium dependent. On the other hand, MLT induced intracellular calcium release by colostrum phagocytes. Some authors have shown that TMB-8 effects include inhibition of calcium influx but not calcium release [56], whereas others reported that TMB-8 inhibits calcium release [57].

The actions and effects of MLT were shown to be transmitted via G-protein-coupled receptors (GPCRs), a class of membrane receptors expressed at low levels in numerous organs and cell types [58], and general dependent on intracellular Ca^2+^ [21].

The microbicidal activity promoted by MLT and the resulting oxidation products may have important clinical implications [59]. The superoxide may come from a change of responsiveness to intracellular Ca^2+^ level and to phosphorylation events during oxidative metabolism [60]. Here we show that MLT is capable of significantly raising intracellular calcium by colostrum phagocytes from normoglycemic women. Therefore, the results supported that melatonin change the intracellular Ca^2+^ level, which facilitate the microbicidal activity of cells.

MLT may influence the activity of phagocytes through changes in the intracellular Ca^2+^ [46]. In this study, in colostral phagocytes form diabetic mothers, the intracellular Ca^2+^ response to MLT stimulation was significantly lower. Although colostral phagocytes from diabetic women were inhibited by MLT, the luzindole treatment also decreased intracellular Ca^2+^ release. Because MLT is known to exert an effect not only via membrane-bound but also nuclear receptors [16) probably the luzindol might not have entirely inhibited the effect of MLT on colostral phagocytes from diabetic mothers.

At physiological concentration, the pineal hormone MLT can stimulate the natural immunity, an important defense with anti-infectious and microbicidal actions [7], [8], [9], [14], [30]. Despite high concentrations of MLT present in colostrum of diabetic mothers colostral phagocytes of these mothers, however, were not stimulated by MLT. This hormone contained in hyperglycemic maternal milk can contribute to protecting the gastrointestinal mucosa of newborns. The action of MLT in the newborn's intestine may be associated to prevention of gastrointestinal mucosa ulceration by antioxidant action, reduction of hydrochloric acid secretion, and immune system stimulation, fostering epithelial regeneration and increased microcirculation [26], especially in infants of diabetic mothers.

## Conclusions

The results obtained suggest an interactive effect of glucose metabolism and MLT on colostral phagocytes. In colostral phagocytes from normoglycemic mothers, MLT likely increases the ability of colostrum to protect against EPEC and other infections. In diabetic mothers, because maternal hyperglycemia modifies the functional activity of colostrum phagocytes, melatonin effects are likely limited to anti-inflammatory processes, with low superoxide release and bactericidal activity.
